# Personalized and long-term electronic informed consent in clinical research: stakeholder views

**DOI:** 10.1186/s12910-021-00675-7

**Published:** 2021-07-31

**Authors:** Evelien De Sutter, Pascal Borry, David Geerts, Isabelle Huys

**Affiliations:** 1 Clinical Pharmacology and Pharmacotherapy, Department of Pharmaceutical and Pharmacological Sciences, KU Leuven, Leuven, Belgium; 2 Centre for Biomedical Ethics and Law, Department of Public Health and Primary Care, KU Leuven, Leuven, Belgium; 3 Meaningful Interactions Lab, KU Leuven, Leuven, Belgium

**Keywords:** Interactive consent, Qualitative research, Tailored, Digital communication, Software tools

## Abstract

**Background:**

The landscape of clinical research has evolved over the past decade. With technological advances, the practice of using electronic informed consent (eIC) has emerged. However, a number of challenges hinder the successful and widespread deployment of eIC in clinical research. Therefore, we aimed to investigate the views of various stakeholders on the potential advantages and challenges of eIC.

**Methods:**

Semi-structured interviews were conducted with 39 participants from 5 stakeholder groups from across 11 European countries. The stakeholder groups included physicians, patient organization representatives, regulator representatives, ethics committee members, and pharmaceutical industry representatives, and all were involved in clinical research. Interviews were analyzed using the framework method.

**Results:**

Interviewees identified that a powerful feature of eIC is its personalized approach as it may increase participant empowerment. However, they identified several ethical and practical challenges, such as ensuring research participants are not overloaded with information and offering the same options to research participants who would prefer a paper-based informed consent rather than eIC. According to the interviewees, eIC has the potential to establish efficient long-term interactions between the research participants and the research team in order to keep the participants informed during and after the study. Interviewees emphasized that a personal interaction with the research team is of utmost importance and this cannot be replaced by an electronic platform. In addition, interviewees across the stakeholder groups supported the idea of having a harmonized eIC approach across the European Member States.

**Conclusions:**

Interviewees reported a range of design and implementation challenges which needs to be overcome to foster innovation in informing research participants and obtaining their consent electronically. It was considered important that the implementation of eIC runs alongside the face-to-face contact between research participants and the research team. Moreover, interviewees expect that eIC could offer the opportunity to enable a personalized approach and to strengthen continuous communication over time. If successfully implemented, eIC may facilitate the engagement of research participants in clinical research.

**Supplementary Information:**

The online version contains supplementary material available at 10.1186/s12910-021-00675-7.

## Background

The informed consent (IC) process in clinical research refers to informing a potential research participant of all the pertinent aspects of a clinical study by presenting the information in a IC document [1]. However, lengthy and complex paper-based IC documents have been proven to be ineffectual in providing information in an understandable manner to research participants [2, 3]. As a result, researchers have attempted to make the IC process more engaging and informative [4]. Presently, there is a popular shift towards using digital technologies. Available research shows that more than 80% of clinical research sponsors will implement electronic informed consent (eIC) in the short term [5].

eIC is regarded as a promising alternative to paper-based IC in clinical research [6, 7]. For instance, by offering tailored information based upon the preferences of research participants, it places them centrally and this may result in a more active engagement [6, 8]. Moreover, eIC could enable a long-term interaction with research participants by facilitating ongoing communication with the research team during and after a research study [8]. This interaction with research participants may be beneficial for informing the participants of updates to the IC due to new research requirements, or other unforeseen circumstances, or to provide them with research results [6]. Throughout this manuscript, eIC refers to an interactive online-based IC application which could facilitate interactions over time and could enable a personalized approach, adapted to research participants’ needs.

Within the European Union, the conduct of clinical research is governed by a large number of legal instruments, such as the Clinical Trials Directive 2001/20/EC (CTD) which will soon be replaced by the Clinical Trials Regulation 536/2014 (CTR) [9–11]. Moreover, the General Data Protection Regulation 2016/679 (GDPR) lays down the foundation to protect research participants’ rights in relation to the processing of their personal data [12]. Under the CTD and the CTR, written IC needs to be obtained for participation in clinical research, while the GDPR allows a written statement, including in electronic form, to obtain research participants’ IC for data processing [9, 11, 12]. Moreover, the Regulation on electronic identification and trust services for electronic transactions in the internal market (eIDAS Regulation) describes that “*a qualified electronic signature shall have the equivalent legal effect of a handwritten signature*” [13]. However, this Regulation is not specifically focused on obtaining participants’ eIC in the context of clinical research [13, 14]. Therefore, compliant use of eIC to convey information and to obtain research participants’ consent is a matter of ongoing debate. National laws concerning the use of eIC in clinical research are highly variable across European countries [14]. Some national laws do not mention eIC; for example, in Spain or Finland. Examples of the legal acceptance of eIC in some countries included in this manuscript are presented in Table 1.

**Table 1:** Examples of the legal acceptance of eIC in the context of clinical research participation

European country	Is it legally allowed to use eIC in clinical research?
Austria	eIC can be used in clinical trials [15]
Belgium	A guidance document was developed by a working group on IC, specifying the needs and restrictions when using eIC in interventional clinical trials [16]
The Netherlands	The Netherlands allow only a paper-based signature. Nevertheless, the Medical Research Involving Human Subjects Act is currently under revision to include the use of electronic signatures [17]
The United Kingdom	The Health Research Authority and the Medicines and Healthcare products Regulatory Agency published a joint statement on the use of eIC in clinical research [18]

Next to the uncertainties regarding compliant use of eIC, a number of other issues remain that are important to resolve in order to achieve the trustworthy implementation of eIC in clinical research. Specifically, there is a need for structured insights regarding how an eIC platform (through which research participants are able to manage their eIC) may be effectively personalized and also on how the long-term interactions may be established. A complete overview of the aspects of an eIC platform that are acceptable to personalize is currently lacking [19]. With regard to the long-term interactions, it is necessary to determine which results may be shared with the research participants via eIC. In addition, the impact of eIC on the clinical research process, as well as on the review processes of ethics committees (ECs) and regulators, needs to be addressed. Moreover, the management of an eIC platform requires clarification. The hosting party will be responsible for the correct storage of data and access control and thus, needs careful consideration. This study aims to investigate these issues by collecting the views of multiple stakeholders: physicians, patient organization representatives, regulator representatives, EC members, and pharmaceutical industry representatives. The study has one main research question: What are stakeholders’ views on the potential advantages and challenges of eIC for the current clinical research process?

The insights provided by these different stakeholder groups may support the development and responsible implementation of interactive eIC approaches that will stimulate research participants to be more empowered and informed.

## Methods

This study is reported in accordance with the Consolidated Criteria for Reporting Qualitative Research (COREQ) checklist (Additional file 1) [20].

### Approach

Semi-structured interviews were conducted to seek the views and experiences of various stakeholder groups across Europe and the United Kingdom (UK) regarding (electronic) IC in clinical research. An interview guide was developed based upon a systematic literature review and the research aims [19]. This systematic literature review identified that more research regarding the personalization of an eIC platform and the long-term interaction with research participants was required. In addition, several other issues remained, such as those related to the hosting of an eIC platform. Some questions were slightly different for each stakeholder group (Additional file 2). Questions on the current paper-based IC process were included to explore potential strategies for enhancing the implementation of eIC. Specific eIC-related questions were also added. The interview questions were tested in three pilot interviews. The interview guide was provided in advance to interviewees upon request.

### Interviewee selection and recruitment

Semi-structured interviews were conducted with people from 5 stakeholder groups: pharmaceutical industry representatives, patient organization representatives, regulator representatives, EC members, and physicians. Patient organization representatives and physicians involved in different disease areas were recruited. Moreover, we included physicians who work in academic institutions, as well as those who work in the pharmaceutical industry. Interviewees were recruited through purposive sampling and snowballing where existing interviewees suggested potential recruits. Potential interviewees were identified by exploring stakeholder websites and existing literature with regard to eIC or upon referral by the research group’s network. Interviewees were eligible for inclusion when they (i) were active in a European country, (ii) had fluent proficiency in English or Dutch, and (iii) were familiar with (electronic) IC in clinical research. An invitation containing the IC for the interview study was mailed to suitable interviewees. Recruitment continued until data saturation was achieved. In total, 39 interviews were conducted with representatives of the pharmaceutical industry (n = 8), of patient organizations (n = 7), of regulators (n = 5), physicians (n = 6), and EC members (n = 13, of whom n = 4 were also physicians). Interviewees across the stakeholder groups were working in Austria, Belgium, Finland, Germany, Latvia, Lithuania, the Netherlands, Portugal, Slovakia, Spain, the UK, or were active at a pan-European level.

### Data collection

Interviews took place between March and September 2020 and were conducted by using Skype for Business or other electronic means if preferred by the interviewee. All interviews were conducted by the same researcher (EDS). At the start of each interview, the interviewer introduced herself and described the aim of the interview study. Interviewees were always provided with the definition of eIC as issued by the US Food and Drug Administration (FDA) to emphasize that both providing study-related information to participants and obtaining their consent occur via electronic means [21]. Interviews were conducted in Dutch or English and were digitally audio-recorded. The interviews were 20–60 min long. Only the interviewer and the interviewee were present during the interview.

### Data analysis

Interviews were analyzed by applying thematic analysis, according to the framework method [22]. Pseudonymized audio-recordings were transcribed verbatim by a third-party and by the interviewer (EDS). The transcripts were thoroughly read by EDS who made reflective notes related to the content of the transcripts and, if necessary, listened to some parts of the audio-recordings again. Subsequently, NVivo software was used to code the transcripts by applying a combination of an inductive and deductive approach. The latter was based upon themes integrated in the interview questions. The first two transcripts were coded independently by EDS and F. Vanendert (FV) or B. Coopmans (BC). The coded transcripts were compared, and the assigned codes were grouped into broader categories by using a coding tree (Additional file 3). As a result, a working analytical framework was developed. Hereafter, one researcher (EDS), using NVivo, applied this analytical framework to the other transcripts. The coded text was exported from NVivo to Microsoft Excel in order to create a framework matrix. In this matrix, the transcripts were summarized for each code and each interviewee to allow for comparison between and within different stakeholder groups. The data obtained was then interpreted.

## Results

### Current paper-based informed consent process

#### Advantages

Many interviewees indicated that paper-based IC involves a face-to-face conversation with a healthcare professional, such as a physician. They underlined that the face-to-face contact between research participants and the physician is a crucial part of the paper-based IC process. Firstly, it facilitates the verification of research participants’ identity and secondly, the interviewees believed that the presence of a physician is necessary to convey the consent options to the research participants and ensure that they are adequately informed.

A health professional can easily determine from the body language and the eye contact of study participants whether they are really ready to participate or not and whether they still have doubts or questions. (14, EC member)

These interviewees believed that the paper-based IC document was the traditional method of providing information to research participants, and due to this, the paper-based form is manageable for the majority of participants. Moreover, the interviewees agreed that as the paper-based document is a fixed document, it offers the research participants the opportunity to discuss the research study with family members, friends, or a general practitioner. It allows the research participants to review what they have consented to after signing the IC. In addition, paper-based IC enables research participants to highlight certain parts of the document and compose further questions.

#### Challenges

The majority of interviewees across the stakeholder groups recognized that paper IC documents are often very long and complex to read and understand. The information described in the IC often contains a specialized medical and legal terminology. As a result, it is not always clear to the research participants what is expected from them.

There is the issue of the IC being rather lengthy nowadays. Not only understanding, but also reading through everything and concentrating on everything might be more of an issue, maybe even more than understanding the document. (4, regulator representative)

Nevertheless, all EC members asserted that the reading level of the IC document is reviewed when assessing a research protocol. Their assessment of the language used is based upon their own experiences or perspectives, and they do not have concrete tools to easily identify the readability of the information.

Another challenge raised by the interviewees was related to ensuring the documentation was correct and to the storage of the paper-based IC. Archiving was considered challenging for both the research participants and the research sites due to repository and document retrieval difficulties. Several physicians and regulator and pharmaceutical industry representatives indicated that paper-based IC is associated with various documentation issues, such as signatures on incorrect IC versions or the erroneous placing of signatures on the document.

Several interviewees remarked that a paper-based IC is static and, thus, is not tailored to the needs of the research participants.

Patient preferences at one particular time will not necessarily be the same throughout a study and the only way for patients to change their mind during a study is to withdraw from it rather than to change an option. (3, physician)

Moreover, it was argued that establishing a long-term interaction between research participants and the research team is complicated. It was considered to be burdensome for the research team if the need arose to recontact research participants to obtain reconsent or present a lay summary. It also placed time and logistical demands on the research participants to make additional visits to the research site in order to sign new versions of the IC document.

### Electronic informed consent process

The majority of interviewees reported they did not have experience with eIC; however, a few interviewees have gathered practical experience or have been involved in theoretical discussions about eIC. If relevant, we have indicated which answers stemmed from experienced interviewees.

#### Personalization

##### Functionalities

When interviewees were asked how they would personalize an eIC platform, several functionalities were suggested (Table 2).

**Table 2:** Stakeholders’ proposals regarding the functionalities of a personalized eIC platform

Present a first layer of information that is essential for participation in the research study, followed by a second layer that offers more specific information to those who are interested (e.g., by using hyperlinks or by hovering over a word)
Enable the research participants to indicate if, how, and for which reasons they would like to be recontacted. For instance, for receiving information regarding the status of the research study, the results, amendments, studies that will take place in the coming months, etc
Enable the research participants to change the layout (e.g., font size) and highlight information
Provide research participants with the option to choose between audio or video content (particularly in case of hearing or visual impairment)
When implementing a quiz to assess research participants’ comprehension, automatically redirect them to the relevant information/specific topic when they have answered incorrectly
Provide the possibility to implement metrics that allow physicians to monitor various variables (e.g., how long did it take for research participants to read a certain topic)
Offer the ability for physicians to stratify groups and provide adapted information (e.g., only give information about pregnancies and breastfeeding to women with a childbearing age)

##### Advantages of a personalized approach

Some interviewees stated that a personalized approach is the strength of eIC. They mentioned that eIC enables research participants to adapt how they would like to receive information about the research study based on their preferences.

As it concerns voluntary participation, research participants need to have the possibility to indicate their preferences. (2, physician)

Some EC members and regulator and patient organization representatives highlighted that a personalized approach may partly solve the issue of flooding the participants with a large amount of information by adapting the information to the needs of the participants.

I think a tiered approach is a very good option as there is very often too much information in the consent form, so that it sometimes really makes the most important information obscure. (4, regulator representative)

Furthermore, some interviewees believed that a personalized approach is very valuable for both the research participants and the physicians. Research participants are able to receive tailored information increasing their empowerment and enabling more focused conversations with the physician. Personalization also stimulates individuals to be more engaged and better informed according to their needs.

A personalized approach would be more tailored to the particular situation of research participants. I think it would help to understand the information. (3, regulator representative)

##### Challenges of a personalized approach

Several regulator representatives had reservations about personalized eIC platforms. They stated that, from a legal point of view, every potential participant needs to receive all the relevant information pertaining to a research study in order to make an informed decision. Many other interviewees across the stakeholder groups agreed and mentioned that it must not be allowed that some participants have access to more information than other participants. Personalization can also be an issue when both a paper-based IC and eIC are offered during a specific research study. Research participants receiving a paper-based IC should also have the opportunity to read the additional information. Another concern that was raised related to the amount of additional information that participants could choose to receive.

You need to make sure that the research participants will not be overwhelmed with information. (4, physician)

Moreover, questions arose regarding how to present information in a correct and tailored manner to the research participants. According to EC members and pharmaceutical industry and regulator representatives, it would be challenging and time-consuming to develop such a personalized eIC platform. To make it feasible, one pharmaceutical industry representative advised that a template for a personalized eIC platform, which already offers the different functionalities, be developed. The pharmaceutical industry representatives also thought that it would be challenging for ECs to review a personalized eIC platform.

Some interviewees mentioned that not all research participants are able to manage the responsibility of indicating their preferences themselves. One physician raised the point that if the research participant has too much responsibility and freedom, it could affect the quality of the study. Another physician, having had practical experience with an eIC platform, stated that personalization complicates the IC process because verification of who has given permission for which aspects is required. However, this may be done automatically.

In a modern age, checking the preferences of the participants is done electronically by using automated pathways. (3, physician)

#### Long-term interaction

Stakeholders agreed that eIC offers the possibility to establish a long-term interaction between research participants and the research team. One of the abovementioned functionalities of a personalized eIC platform states that the decision to maintain a longitudinal relationship with the research team lies with the research participant. Moreover, it was agreed that eIC can facilitate continued contact with participants for research purposes as electronic devices were considered to be a convenient method of approaching participants. It was believed that the potential to recontact participants easily is valuable as research is dynamic in character and, therefore, it is not always feasible to anticipate all of the research requirements in advance. One pharmaceutical industry representative argued that being able to recontact participants easily would be beneficial to scientific research.

It would be an advantage for scientific research. There would be more flexibility in what you can do with already collected data. (2, pharmaceutical industry representative)

##### Type of communication

Interviewees raised the point that the long-term interaction may be used to inform the research participants of various aspects of the research study. Two patient organization representatives asserted that patients sometimes do not know, or remember, that they are participating in a research study. Therefore, they advised that repeated IC discussions are set up or that feedback is provided from the study center on regular basis. Similarly, an EC member also voiced the opinion that it would be valuable to set up a repeated IC discussion, particularly if it concerns a long-term study. Contact with the participants may also be maintained in order to inform them of updates to the IC. As a result, the participants are not necessarily required to go to the research site physically in order to sign a new version of the IC. Information regarding the status of the research study or the various steps involved in the research process may also be shared with the research participants. It was highlighted that this information would be valuable to inform participants about the different phases and the duration of the drug lifecycle to avoid any misunderstanding.

Lay persons usually have no idea how long it takes to develop a drug and to get it on the market. Sometimes they have the feeling that there is one study and when the study is successful, the medication will be available tomorrow. (11, EC member)

**Fig. 1:**
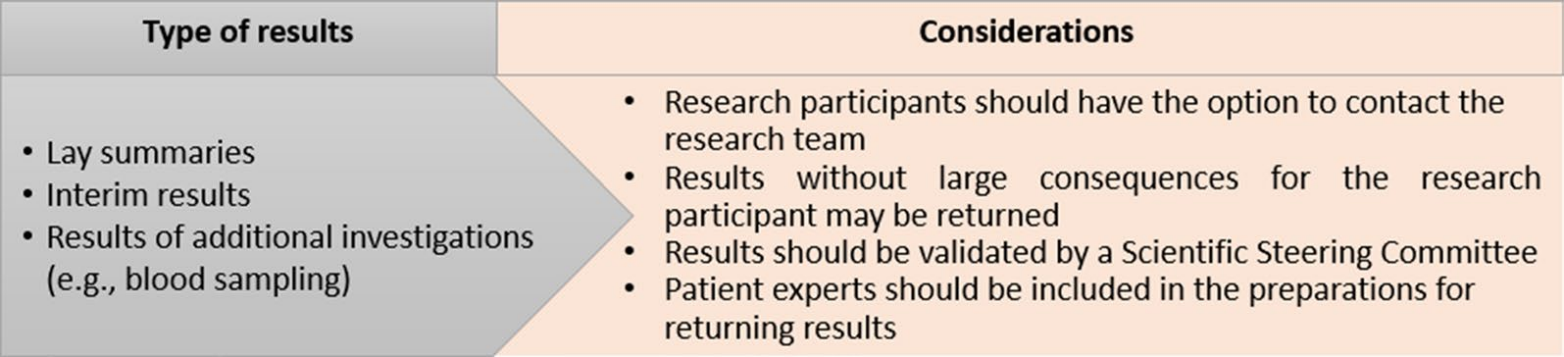
Stakeholders’ views on the type of results that could be returned and their considerations

An eIC platform may also be used to return the results of the research study to the participants. The views of the various stakeholders about the type of results that may be returned and their considerations on this matter are shown in Fig. 1. Several patient organization representatives stated that research participants have the right to receive the results of the research study in which they have taken part. Therefore, they advocated that it should be up to the research participants to decide which type of results they would like to receive through the eIC platform. Nevertheless, one physician and one regulator representative stated that it is not appropriate to return results via an electronic device. They were convinced that a clinical consultation is necessary to convey these results to the research participants. Other interviewees indicated that information not related to serious health conditions of the research participants may be shared easily via the eIC platform. However, research participants should be able to contact the research team if they have questions and require further explanation.

An electronic platform is not an excuse to not give participants the information they need, and which they need face-to-face. (3, pharmaceutical industry representative)

Moreover, some interviewees were convinced that an institutional policy, discussed and agreed upon by all relevant stakeholders, should be established to prevent differing practices of returning information from study to study.

Several interviewees were of the opinion that information needs to be returned in an understandable and participant-friendly manner in order to prevent harming the participants. Therefore, it was argued that a deeper reflection of which results may be returned and how this information may be interpreted by a lay person is needed. One patient organization representative advocated for a patient expert to be included when preparing for the return of information.

#### Types of clinical research studies

**Fig. 2:**
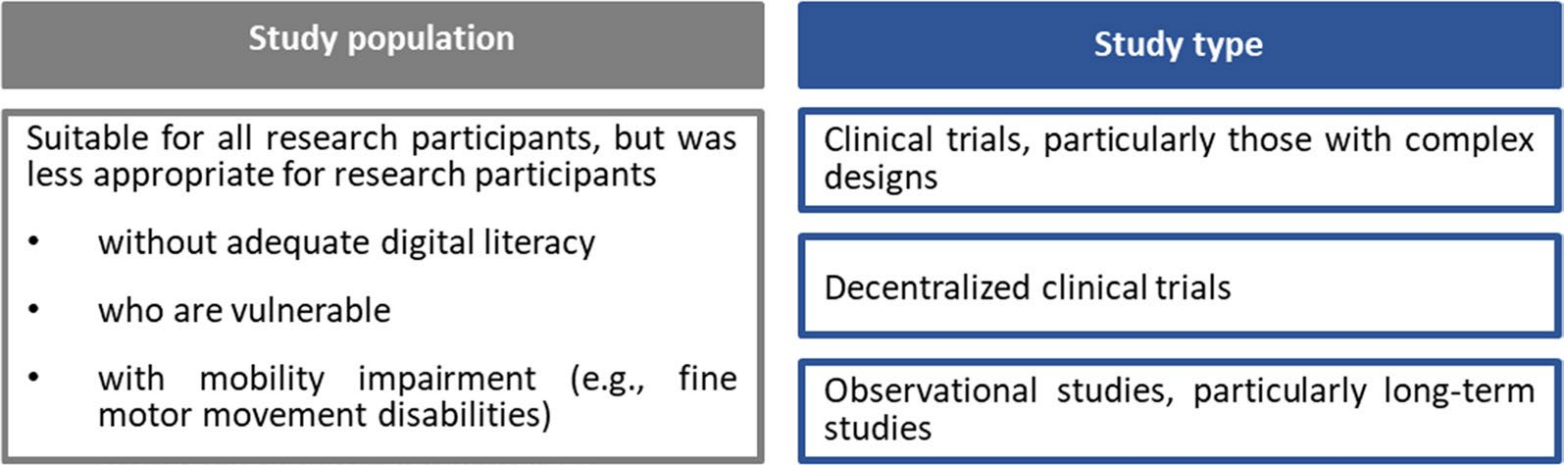
Stakeholders’ views on the use of eIC in clinical research

Interviewees agreed that eIC may be implemented quite broadly. The views of the stakeholders on the study population and the study type for which eIC may be used are presented in Fig. 2. In addition, it was mentioned that eIC is beneficial for conducting research during the COVID-19 pandemic as it reduces visitations to research sites.

Interviewees raised the point that research participants must not be excluded from a particular clinical research study due to a lack of digital literacy. Therefore, they advocated for research participants to always be offered the possibility to read and sign a paper-based IC instead of an eIC. It was considered important to promote participation, particularly for those with unmet medical needs, as participation in a clinical study may be the last opportunity for some research participants to improve their health-related condition.

The physicians and pharmaceutical industry representatives, who already have had experience with eIC, mentioned that they used eIC in different study populations and therapeutic areas. Moreover, one pharmaceutical industry representative indicated that eIC was rapidly being accepted by research participants.

#### Impact on clinical research studies

##### Recruitment, dropout, and understanding

The perspectives of the interviewees regarding the impact that eIC could have on recruitment, dropout, and understanding are presented in Table 3.

**Table 3:** Perspectives of stakeholders on the potential impact of eIC on recruitment, dropout, and understanding

Recruitment	Dropout	Understanding
Positive impact	Positive impact	Positive impact
Would be facilitated, especially for rare diseases	Frequent updates could keep participants interested	Interactive tools could facilitate comprehension and retention of knowledge
More diverse population	Participants could have a better understanding of what to expect	Implementation of metrics could support physicians when explaining the research study

Negative impact	Negative impact	Negative impact
Could widen the digital gap	Electronic systems could be too complicated	Information overload
Would be difficult to establish a relationship of trust	Participants may stop participating due to less personal contact	

Various interviewees were of the opinion that the implementation of eIC in clinical research studies could have both a positive and a negative impact on the recruitment and dropout of research participants. However, one pharmaceutical industry representative, already having had experience with eIC, noted that the impact on recruitment and dropout is difficult, or time-consuming, to quantify. Similarly, interviewees expected that eIC could have both a positive and negative impact on the understanding of research information. Nevertheless, two EC members and one physician were of the opinion that eIC would not impact understanding.

I do not see the difference of understanding the information between eIC and the paper-based IC form. If there are problems to understand, they will occur in both formats. (13, EC member)

Interviewees posed the notion that the use of eIC could have a negative influence on the contact between the physician and research participants, and this could adversely affect recruitment and drop-out. They emphasized that an electronic platform cannot replace the personal interaction between the physician and the research participant. However, they mentioned that eIC can supplement the oral explanation given by the physician. Moreover, it was mentioned that the decision of a research participant to take part in a study is sometimes based upon a relationship of trust that is developed with the physician, which may be more easily established through personal contact.

The process of informing the participant should be more or less the same as with paper-based. (2, regulator representative)

##### Documentation and storage

All stakeholder groups agreed that using eIC will probably improve the process of signing and dating IC. eIC has the potential to disable errors, such as recording an incorrect date, and therefore, it will support quality assurance. Interviewees also believed that it is easy to securely store eIC, for example by enabling password protection. However, interviewees across stakeholder groups identified that the primary challenge of implementing eIC is the protection of the research participants’ privacy during storage. It was mentioned that eIC platforms need to be adequately secure in order to avoid confidentiality infringements. Moreover, one regulator representative thought that European servers should be used to store the data, preferably in the country where the data is collected and used.

Regarding inspections, the regulator representatives stated that they must be able to track who has handled the data. In addition, they would thoroughly investigate the eIC platform during inspections in order to assess if it could damage the rights of the research participants. To end, they mentioned that they will require proof that the eIC will be accessible for at least 25 years, as is stated in the CTR.

### Impact review processes

EC members and regulator representatives believed that the use of eIC platforms may slow down their processes of reviewing study protocols initially, particularly when eIC platforms become widely employed. They mentioned that they would have to consider additional aspects compared to those of the paper-based IC. For example, proof regarding compliance of electronic signatures with the applicable legislation, or a statement confirming face-to-face contact between the participant and physician, would be required. However, EC members and regulator representatives stated that if a robust platform, checked by an independent party, was used, then the ECs could immediately trust the platform, and this would result in an accelerated assessment.

One unique platform will streamline the assessment. If the platform is already validated and accepted by regulators then it would be quite easy. (3, regulator representative)

Nevertheless, it was strongly corroborated that guidelines would be useful to determine the quality criteria for an eIC platform and this would facilitate the reviewing process.

Two EC members, who already had developed experience with reviewing an eIC platform, reported different views. The first EC member argued that an eIC platform was challenging to review and control, whereas the second EC member did not experience major differences from the review process of a paper-based IC. Several pharmaceutical industry representatives acknowledged that it is not clear how they should submit eIC to ECs and whether the ECs would like to have access to the platform. In addition, one EC member mentioned that ECs in several countries are independent, and this could have a negative impact on a harmonized review process.

A more centralized approach regarding ECs would be better. Then you could have a library with approved eIC material and that would be very helpful. (7, EC member)

#### Hosting an eIC platform

Interviewees who have had previous practical experience with eIC platforms, used those offered by private vendors or academic institutions. The other interviewees reported differing opinions on who should be responsible for hosting an eIC platform; however, it was widely agreed that it should not be the sponsor. Three possibilities for eIC platform hosts arose out of their responses. The first was that a regulatory body should host the platform, preferably at a European level.

There would be variations of the platform in each country, unless it is agreed at the European level. It could be hosted by a centralized body such as the European Medicines Agency and you could have a single identical platform in all countries. (3, regulator representative)

The second possibility was that an eIC platform be hosted by the principal investigator, as he or she was already responsible for managing the paper-based IC documents. Nevertheless, the investigator would need to have a ready-to-use platform to ease the administrative burden.

The third possibility was that a trusted center, controlled by a regulatory body, hosts the platform. It was considered crucial that it be a center that would hold the participants trust.

Moreover, managing an eIC platform goes hand in hand with its financing. For all of the abovementioned possibilities, it was suggested that the hosting of an eIC platform be financed independently.

#### Towards harmonization

Several interviewees advocated for the development of a European eIC platform. It was mentioned that when a research site conducts multiple clinical research studies at the same time, it would be more user-friendly to use a single platform. Moreover, one pharmaceutical industry representative believed that eIC platforms created by commercial vendors will never be accepted by all the clinical research stakeholders. Also, it would be very challenging for physicians if there were numerous eIC platforms.

When physicians would participate in 5 clinical trials at the same moment, they would have to adjust themselves to these different platforms. (9, EC member)

Nevertheless, the interviewees raised several challenges related to this harmonized European approach. First, the legal requirements between Member States differ. For example, some Member States still require a wet-ink signature for study participation. Second, the development of IT in the various research sites or hospitals within each Member State differ. Interviewees noted that eIC could be integrated into established systems such as e-Health. Nevertheless, these systems may not always support the integration of eIC. Third, future participants, as well as other stakeholders involved in clinical research, need to accept this single European eIC platform. For example, sponsors may prefer to retain their own method of collecting IC or have already invested in eIC platforms.

Moreover, pharmaceutical industry representatives stressed the need for a European guidance for the implementation and use of eIC in clinical research. They were convinced that one of the reasons holding back the adoption of eIC is the lack of such a guidance. This guidance could provide a framework for harmonization across European Member States.

We are conducting trials in 20 European countries. It is not feasible to have different eIC systems for these countries. (5, pharmaceutical industry representative)

## Discussion

This manuscript presents the opinions of various stakeholders regarding the potential advantages and challenges of eIC. Whereas previous research has mostly focused on investigating potential opportunities of eIC or assessing participants’ understanding when using eIC, this study has taken a more precise approach on how an eIC platform can be effectively personalized and how the interaction over time can be guaranteed. Therefore, this study adds to the scarce empirical literature on personalization and the longitudinal interaction in the context of eIC. In addition, this study identified interviewees’ divergent views with regard to the hosting party of an eIC platform and the impact of eIC on the clinical research process. The various key findings that emerged from this study need to be considered when designing or implementing eIC in clinical research.

### Enhancing the IC experience

Several interviewees highlighted that paper-based IC documents are difficult to understand due to the scientific and legal nature of the information and emphasized the importance to present information in an understandable and engaging manner to research participants. eIC can assist in this by making use of video, audio or graphics to convey information to participants, which is considered more effective than written text [19]. Nevertheless, a small number of interviewees were convinced that eIC would not influence the understanding of research participants compared to that of a paper-based IC. Similarly, other research has shown that eIC does not necessarily result in the enhanced understanding of research participants [4]. Therefore, in addition to multimedia interventions, more needs to be achieved in order to support a more readily understandable eIC. Efforts should be directed to involve patients in the design of eIC. For example, Ramos et al. involved patients to design and test an eIC interface. In this way, patients can provide feedback on several aspects of this interface, such as the ease of use or the presentation of information [23]. Moreover, a widely accessible library could be established that will store multimedia elements which present information, not related to a specific study [24]. This library could be used for designing eIC in a participant-friendly way. Interviewees agreed that eIC could be used in a wide range of clinical research studies. However, research participants should always be given the choice between eIC and a paper-based IC. If eIC would be used remotely, participants can prefer a paper-based IC because they lack digital literacy or do not have access to electronic devices. However, offering both eIC and paper-based IC may increase the workload of the researchers or the sponsor.

### Personal contact

Personal contact between the research participant and physician was valued highly. According to some interviewees, the decision of a potential participant to partake in a research study relies on a relationship of trust developed with the physician, which is in line with the available literature [25, 26]. Dellson et al. showed that the decision of patients to participate in a clinical trial is guided by the doctor-patient relationship and the trust that they have in the physician rather than by the information provided in the IC document [25]. Interviewees were of the opinion that using eIC would negatively impact personal contact. However, a review found that the implementation of digital health technologies, such as eIC, may result in increased personal contact between the research participant and physician. If eIC would result in a more efficient way of working for physicians, they have more time to inform participants and to answer their questions [27].

### An interactive consent interface

Interviewees widely agreed that eIC can support the establishment of long-term interactions between the research team and research participants. Some interviewees suggested that these long-term interactions could be used to continuously educate participants. For example, the study requirements, that participants need to comply with, may be emphasized by having repeated IC discussions [28]. The strengthening of continuous communication with participants during a research study may have a positive impact on participant compliance with these requirements [3]. Additionally, interviewees mentioned that an eIC platform can also be used to provide generalized or individual results related to the research study. However, concerns were reported about how the results would be interpreted by lay individuals. Therefore, when using eIC, it is advisable to involve patient representatives who are able to develop or review the information that will be returned to research participants [29].

In addition, some interviewees indicated that a personalized eIC platform could empower research participants. Nevertheless, several obstacles related to personalization were discussed. For example, it was acknowledged that caution should be taken to not overwhelm research participants. Research has found that overloading research participants with information can impact their capabilities regarding making healthcare decisions [30]. Therefore, the structure for how information is presented is important; for example, switching between brief and detailed content. Information may be offered in layers: a first layer offering essential information, followed by a second layer that allows research participants to access additional information if preferred [24]. An interactive eIC system allows research participants to indicate how they would like to be involved. However, interviewees indicated that not all research participants are able to set their own preferences. Some research participants may not have the capacity to make sound health-related decisions as their health literacy is limited [31]. Moreover, when too much responsibility is given to research participants, who may be facing other challenges, there is a risk that they feel too pressured to adapt an eIC platform based on their preferences.

### A harmonized eIC approach across European Member States

Interviewees supported harmonization across European Member States and beyond regarding eIC. However, they indicated that establishing a harmonized approach would be challenging. For example, some European Member States, such as Hungary, are reluctant to use eIC in clinical trials [32]. Several interviewees showed a preference for developing and using a European eIC platform. This would benefit EC members and regulator representatives when assessing study protocols or conducting inspections. Nevertheless, there were divergent views about who should manage this platform. Interviewees expressed confidence in regulatory bodies, physicians, and trusted centers to perform the role of hosting party. Moreover, the management and financing of a European eIC platform cannot be considered separately. For example, there are costs associated with maintaining the system as well as with the personnel responsible for controlling the access of the different user groups.

### Study limitations

Although this qualitative study provides valuable information for the implementation of eIC, it has some limitations. Qualitative research does not allow for the generalization of results. Moreover, a self-selection bias may have been introduced because it is possible that participants who are more interested in eIC agreed to take part in this study. It is also important to recognize that the majority of interviewees did not have practical experience with eIC, and therefore, some questions remained theoretical. However, in the event that eIC was implemented in the daily practice of the stakeholders, these views may change. Nevertheless, this qualitative study identified the perception of interviewees without practical experience regarding eIC.

## Conclusions

This qualitative study indicates that stakeholders expect several advantages to a personalized eIC platform that enables long-term contact between researchers and research participants. However, for effective and widespread eIC implementation, several challenges need to be overcome. Attention needs to be paid to the preferences of the research participants regarding contact with the research team and the choice between eIC or a paper-based IC. Moreover, interviewees expressed the wish to establish a harmonized eIC approach across the European Member States.

## Supplementary Information

**Additional file 1:** COREQ checklist

**Additional file 2:** Interview guide

**Additional file 3:** Coding tree

## Data Availability

The datasets generated and/or analyzed during the current study are not publicly available due to confidentiality requirements and research participants who did not provide consent to make their transcripts publicly accessible but are available from the corresponding author on reasonable request.

## Abbreviations

COREQ: Consolidated Criteria for Reporting Qualitative Research
CTD: Clinical Trials Directive 2001/20/EC
CTR: Clinical Trials Regulation 536/2014
EC: Ethics committee
eIC: Electronic informed consent
FDA: US Food and Drug Administration
GDPR: General Data Protection Regulation 2016/679
IC: Informed consent
UK: United Kingdom
