# Supplementary material for: Personalized and long-term electronic informed consent in clinical research: stakeholder views
Source: BMC Med Ethics. 2021 Jul 31;22:108. doi: 10.1186/s12910-021-00675-7 (PMC8325412; doi:10.1186/s12910-021-00675-7)
Supplement: Supplementary file 1 — Additional file 1: COREQ checklist [file 12910_2021_675_MOESM1_ESM.docx]

**Personalized and Long-term Informed Electronic Consent in Clinical Research: Stakeholder Views**

Evelien De Sutter^1^, Pascal Borry^2^, David Geerts^3^, Isabelle Huys^1^

^1^Clinical Pharmacology and Pharmacotherapy, Department of Pharmaceutical and Pharmacological Sciences, KU Leuven, Leuven, Belgium

^2^Centre for Biomedical Ethics and Law, Department of Public Health and Primary Care, KU Leuven, Leuven, Belgium

^3^Meaningful Interactions Lab, KU Leuven, Leuven, Belgium

## Additional file 1: COREQ checklist

| **Item and guide questions** | **Response** | **Location in manuscript (Section)** |
| --- | --- | --- |
| **Domain 1: Research team and reﬂexivity** | | |
| **Personal Characteristics** | | |
| 1. Interviewer/facilitator:  Which author/s conducted the interview or focus group? | EDS | Methods |
| 2. Credentials:  What were the researcher’s credentials? E.g., PhD, MD | PharmD | - |
| 3. Occupation:  What was their occupation at the time of the study? | At the time of the study, EDS working towards her PhD at KU Leuven | - |
| 4. Gender:  Was the researcher male or female? | Female | - |
| 5. Experience and training:  What experience or training did the researcher have? | EDS had previous experience in conducting interviews | - |
| **Relationship with participants** | | |
| 6. Relationship established:  Was a relationship established prior to study commencement? | Prior to the interview, participants were briefed on the study and were provided with the informed consent | Methods |
| 7. Participant knowledge of the interviewer:  What did the participants know about the researcher? | The participants were informed that EDS was researching electronic informed consent as part of her PhD | Methods |
| 8. Interviewer characteristics:  What characteristics were reported about the interviewer/facilitator? | The background of EDS and that this interview study was part of her PhD | Methods |
| **Domain 2: Study design** | | |
| **Theoretical framework** | | |
| 9. Methodological orientation and theory:  What methodological orientation was stated to underpin the study? | Semi-structured interviews that were analyzed using a framework method | Methods |
| **Participant selection** | | |
| 10. Sampling:  How were participants selected? | Through purposive and snowballing sampling | Methods |
| 11. Method of approach:  How were participants approached? | Via mail | Methods |
| 12. Sample size:  How many participants were in the study? | 39 participants were interviewed | Methods |
| 13. Non-participation:  How many people refused to participate or dropped out? Reasons? | 71% of invited individuals refused to participate. Reasons included unavailability, non-response to the invitation, or they had insufficient knowledge of the IC process in clinical research | - |
| **Setting** | | |
| 14. Setting of data collection:  Where was the data collected? | Interviews took place remotely | Methods |
| 15. Presence of non-participants:  Was anyone else present besides the participants and researchers? | No | Methods |
| 16. Description of sample:  What are the important characteristics of the sample? | Participants belong to one of the five stakeholder groups. They were located across European Member States and the UK | Methods |
| **Data collection** | | |
| 17. Interview guide:  Were questions, prompts, guides provided by the authors? Was it pilot tested? | Upon request, guides were provided to the interviewee. The interview questions were tested in three pilot interviews | Methods |
| 18. Repeat interviews:  Were repeat interviews carried out? If yes, how many? | No | - |
| 19. Audio/visual recording:  Did the research use audio or visual recording to collect the data? | Digital audio-recordings of all interviews were made | Methods |
| 20. Field notes:  Were ﬁeld notes made during and/or after the interview or focus group? | Yes | Methods |
| 21. Duration:  What was the duration of the interviews or focus groups? | Interviews were 20 to 60 minutes long | Methods |
| 22. Data saturation:  Was data saturation discussed? | No | - |
| 23. Transcripts returned:  Were transcripts returned to participants for comment and/or correction? | No | - |
| **Domain 3: Analysis and ﬁndings** | | |
| **Data analysis** | | |
| 24. Number of data coders:  How many data coders coded the data? | The first two transcripts were coded by two researchers (EDS and FV or BC). The other transcripts were coded by one researcher (EDS) | Methods |
| 25. Description of the coding tree:  Did authors provide a description of the coding tree? | Coding was described in the methods section. Moreover, the coding tree is reported in the supplementary material accompanying the manuscript | Methods – Supplementary material |
| 26. Derivation of themes:  Were themes identified in advance or derived from the data? | Transcripts were coded by using a combination of an inductive and deductive approach | Methods |
| 27. Software:  What software, if applicable, was used to manage the data? | NVivo and Microsoft Excel | Methods |
| 28. Participant checking:  Did participants provide feedback on the findings? | No | - |
| **Reporting** | | |
| 29. Quotations presented:  Where participant quotations presented to illustrate the themes/findings? Was each quotation identified? | Yes | Results |
| 30. Data and ﬁndings consistent:  Was there consistency between the data presented and the findings? | Yes | Results |
| 31. Clarity of major themes:  Were major teams clearly presented in the findings? | Results are presented per major theme | Results |
| 32. Clarity of minor themes:  Is there a description of diverse cases or discussion of minor themes? | Minor themes are discussed in the manuscript, for example by using quotations | Results |
