# Supplementary material for: Personalized and long-term electronic informed consent in clinical research: stakeholder views
Source: BMC Med Ethics. 2021 Jul 31;22:108. doi: 10.1186/s12910-021-00675-7 (PMC8325412; doi:10.1186/s12910-021-00675-7)
Supplement: Supplementary file 2 — Additional file 2: Interview guide [file 12910_2021_675_MOESM2_ESM.docx]

**Personalized and Long-term Informed Electronic Consent in Clinical Research: Stakeholder Views**

Evelien De Sutter^1^, Pascal Borry^2^, David Geerts^3^, Isabelle Huys^1^

^1^Clinical Pharmacology and Pharmacotherapy, Department of Pharmaceutical and Pharmacological Sciences, KU Leuven, Leuven, Belgium

^2^Centre for Biomedical Ethics and Law, Department of Public Health and Primary Care, KU Leuven, Leuven, Belgium

^3^Meaningful Interactions Lab, KU Leuven, Leuven, Belgium

## Additional file 2: Interview guide

1. **Introductory question**
2. To what extent do you have experience with clinical research?
3. **Questions regarding the paper-based informed consent process**
4. What is your experience with the paper-based informed consent process?
5. What are the positive aspects?
6. What are the negative aspects?
7. Does the ethics committee that you are a member of review the reading level of paper-based informed consent forms? *(for ethics committee members only)*
8. **Questions regarding the electronic informed consent process**

Definition (FDA): *“Electronic informed consent refers to the use of electronic systems and processes that may employ multiple electronic media, including text, graphics, audio, video, podcasts, passive and interactive Web sites, biological recognition devices, and card readers, to convey information related to the study and to obtain and document informed consent.”*

1. To what extent do you have experience with electronic informed consent?
2. How did/would electronic informed consent influence the evaluation process? *(for ethics committee members and regulator representatives only)*

The following questions (3-7) apply only to pharmaceutical industry representatives and physicians with practical electronic informed consent experience:

1. In which clinical research study (with regard to the design, population, therapeutic area) do you use electronic informed consent?
2. How does the use of electronic informed consent influence the clinical research process in comparison to a paper-based consent process?
3. How is the electronic informed consent platform designed?
4. Do you have any recommendations? Which one(s)?
5. Does the participant receive personalized information?
6. What is your opinion regarding personalization?
7. What are the advantages and disadvantages of personalization?
8. If relevant, how would you personalize the platform?
9. Who manages the platform?
10. What is your opinion regarding the return of relevant information of a clinical research study to a study participant using the electronic informed consent platform?
11. Which information may be shared with the study participant via the electronic informed consent platform?
12. Who do you think should decide what kind of information may be shared via the electronic informed consent platform?
13. Did you experience any barriers in the implementation of electronic informed consent in this clinical research study/studies? Which one(s)?

The following questions (3-8) apply only to pharmaceutical industry representatives and physicians who do not have experience with electronic informed consent, as well as to patient organization representatives, regulator representatives, and ethics committee members:

1. For which clinical trials (with regard to the design, population, therapeutic area) would electronic informed consent be appropriate?
2. How would the use of an electronic informed consent platform influence the current practice of the clinical research process in comparison to a paper-based consent process?
3. About the design of an electronic informed consent platform:
4. What is your opinion regarding personalization of the platform?
5. What are the advantages and disadvantages of personalization?
6. If relevant, how would you personalize the platform?
7. What is your opinion regarding the return of relevant information of a clinical research study to a study participant using the electronic informed consent platform?
8. Which information may be shared with the study participant via the electronic informed consent platform?
9. Who do you think should decide what kind of information may be shared via the electronic informed consent platform?
10. Which barriers or challenges may be experienced in the implementation of electronic informed consent in clinical research?
11. Who do you think is best placed to manage the platform?

To end *(for all stakeholders)*:

- Do you have any further comments or issues which you think may be relevant to the design, implementation, or use of an electronic informed consent platform?
- Do you have a suggestion for other interested interviewees?
- Do you have any questions?
