# Supplementary material for: Personalized and long-term electronic informed consent in clinical research: stakeholder views
Source: BMC Med Ethics. 2021 Jul 31;22:108. doi: 10.1186/s12910-021-00675-7 (PMC8325412; doi:10.1186/s12910-021-00675-7)
Supplement: Supplementary file 3 — Additional file 3: Coding tree [file 12910_2021_675_MOESM3_ESM.docx]

**Personalized and Long-term Informed Electronic Consent in Clinical Research: Stakeholder Views**

Evelien De Sutter^1^, Pascal Borry^2^, David Geerts^3^, Isabelle Huys^1^

^1^Clinical Pharmacology and Pharmacotherapy, Department of Pharmaceutical and Pharmacological Sciences, KU Leuven, Leuven, Belgium

^2^Centre for Biomedical Ethics and Law, Department of Public Health and Primary Care, KU Leuven, Leuven, Belgium

^3^Meaningful Interactions Lab, KU Leuven, Leuven, Belgium

## Additional file 3: Coding tree

| **Code** | **Sub-code level 1** | **Sub-code level 2** | **Sub-code level 3** | **Description** |
| --- | --- | --- | --- | --- |
| Paper-based IC | Challenges |  |  | What are the disadvantages of the paper-based IC process? |
|  |  | Cognitive burden |  |  |
|  |  | Static |  |  |
|  |  | Long-term interaction |  |  |
|  |  | Documentation and storage |  |  |
|  | Advantages |  |  | What are the advantages of the paper-based IC process? |
|  |  | Traditional |  |  |
|  |  | Fixed |  |  |
|  |  | Face-to-face contact |  |  |
|  | Review reading level |  |  | Does the ethics committee review the reading level of IC? |
| eIC | Experience |  |  | Does the interviewee have experience with eIC? |
|  | Managing the platform |  |  | Who is best placed to manage the eIC platform? |
|  | Impact on review processes |  |  | What is the impact of using eIC on the review processes? |
|  | Types of clinical trials |  |  | For which clinical trials may eIC be used? |
|  |  | Population |  |  |
|  |  | Design |  |  |
|  |  | COVID-19 |  |  |
|  | Personalization |  |  | - What is the opinion of the interviewee on personalization?  - How can an eIC platform be personalized? |
|  |  | Functionalities |  |  |
|  |  | Advantages |  |  |
|  |  | Challenges |  |  |
|  | Long-term interaction |  |  | How can eIC establish a long-term interaction? |
|  |  | Recontacting |  |  |
|  |  | Communication of study results |  | - Which information may be shared via eIC?  - Who should decide what kind of information may be shared via eIC? |
|  |  |  | Decision-maker |  |
|  |  |  | Type of results |  |
|  | Impact on clinical research practice |  |  | How does eIC influence the current process of clinical research? |
|  |  | Understanding |  |  |
|  |  | Recruitment and drop-out |  |  |
|  |  | Documentation and storage |  |  |
|  |  | Face-to-face contact |  |  |
|  | Framework |  |  | How can eIC adoption be facilitated? |
|  |  | Harmonization |  |  |
|  |  | Legal and ethical measures |  |  |
|  | Design of the platform |  |  | Which elements related to the design of eIC are important? |
|  |  | Integration with other systems |  |  |
|  |  | Security |  |  |
